# Bisphosphonates in multicentric osteolysis, nodulosis and arthropathy (MONA) spectrum disorder – an alternative therapeutic approach

**DOI:** 10.1038/srep34017

**Published:** 2016-09-30

**Authors:** Karin Pichler, Daniela Karall, Dieter Kotzot, Elisabeth Steichen-Gersdorf, Alexandra Rümmele-Waibel, Laureane Mittaz-Crettol, Julia Wanschitz, Luisa Bonafé, Kathrin Maurer, Andrea Superti-Furga, Sabine Scholl-Bürgi

**Affiliations:** 1 Clinic for Pediatrics I, Inherited Metabolic Disorders, Medical University of Innsbruck, Anichstrasse 35, A-6020 Innsbruck, Austria; 2 Department of Pediatrics, Division of Neonatology, Pediatric Intensive Care and Neuropediatrics, Medical University of Vienna, Waehringer Guertel 18-20, A-1090 Vienna, Austria; 3 Department of Human Genetics, Medical University of Innsbruck, Peter-Mayr-Strasse 1/1, A-6020 Innsbruck, Austria; 4 Department of Pediatrics, Dornbirn General Hospital, Lustenauerstrasse 4, A-6850 Dornbirn, Austria; 5 Center for Molecular Diseases, Lausanne University Hospital, Av. P. Decker 2, CH-1011 Lausanne, Switzerland; 6 Department of Neurology, Medical University of Innsbruck, Anichstrasse 35, A-6020 Innsbruck, Austria; 7 Department of Radiology, Medical University of Innsbruck, Anichstrasse 35, A-6020 Innsbruck, Austria; 8 Department of Pediatrics, University of Lausanne, Rue du Bugnon 46, CH-1011 Lausanne, Switzerland

## Abstract

Multicentric osteolysis, nodulosis and arthropathy (MONA) spectrum disorder is a rare inherited progressive skeletal disorder caused by mutations in the matrix metalloproteinase 2 (*MMP2*) gene. Treatment options are limited. Herein we present successful bisphosphonate therapy in three affected patients. Patients were treated with bisphosphonates (either pamidronate or zoledronate) for different time periods. The following outcome variables were assessed: skeletal pain, range of motion, bone densitometry, internal medical problems as well as neurocognitive function. Skeletal pain was dramatically reduced in all patients soon after initiation of therapy and bone mineral density increased. Range of motion did not significantly improve. One patient is still able to walk with aids at the age of 14 years. Neurocognitive development was normal in all patients. Bisphosphonate therapy was effective especially in controlling skeletal pain in MONA spectrum disorder. Early initiation of treatment seems to be particularly important in order to achieve the best possible outcome.

Multicentric osteolysis, nodulosis and arthropathy (MONA, MIM #259600) spectrum disorder syndrome is a rare genetic chronic skeletal disorder characterized by multiple peripheral osteolysis, wide metacarpals, osteoporosis, progressive joint contractures, short stature, subcutaneous nodules as well as a coarse face, skin lesions/hirsutism and ocular and cardiac manifestations. Intelligence is normal in the affected patients^1^,^2^,^3^,^4^,^5^,^6^,^7^,^8^,^9^,^10^,^11^. The diagnosis is based on the typical clinical features together with normal laboratory findings, which allow this disease to be distinguished from other syndromes, including fibrotic, rheumatic and lysosomal diseases. MONA spectrum disorder is caused by mutations in the *MMP2* gene (16q12.2) as reported by Martignetti *et al.* in ref. 7. MONA spectrum disorder is considered to be a continuous clinical spectrum involving also Torg, nodulosis, arthropathy and osteolysis (NAO) and Winchester syndrome

The pathogenesis of the disorder remains unclear^12^. To date 43 cases from 26 families with 20 different mutations in the *MMP2* gene have been reported^13^. The enzyme MMP2 belongs to a family of zinc-dependent matrix-degrading enzymes involved in the regulation of angiogenesis^14^,^15^, normal collagen turnover^16^ and tumor cell invasiveness^17^. MMP2 is a major degrader of gelatin and type IV collagen, the major structural component of the basement membrane, as well as elastin, laminin, fibronectin, aggrecan and fibrillin^18^. The actual MMP2 cytogenetic band is 16q12.2.

So far therapy of patients with MONA spectrum disorder has been mainly limited to symptomatic analgesic therapy as well as physical and occupational therapy^12^. Phadke *et al.*^19^ were the first to report beneficial effects of bisphosphonate therapy in MONA spectrum disorder.

In this study we retrospectively analyze the clinical course of three siblings with MONA spectrum disorder over a ten-year period, who were also treated with intravenous bisphosphonates.

## Results

### Characteristics of the patients

The three reported patients are the children of healthy Turkish parents, who were born after uneventful pregnancies. The parents reported not to be related to each other. A fourth male child of the family is healthy.

**Figure 1:**
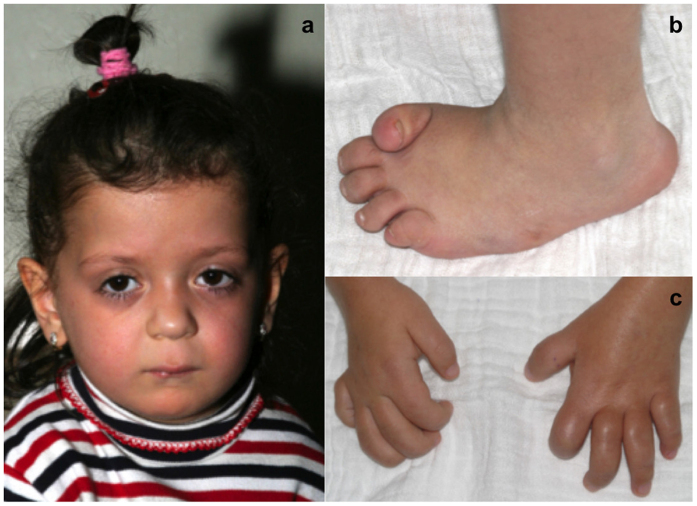
Patient 1 at the age of 4 years, when first symptoms of MONA spectrum disorder developed. She shows mild coarsening of the face (**a**). At the age of 12 years severe flexion contractures were present in both her feet (**b**) and hand joints (**c**).

Patient 1 is the oldest of the reported siblings. She is currently 14 years old and developed painful swelling and contractures of the small hand and foot joints with multiple low-impact fractures, especially of the metatarsal bones, from the age of 3 years on. A coarse face and hypertrichosis but no subcutaneous nodules were present (Fig. 1). Patient 1 was diagnosed with MONA spectrum disorder only 4 years after the symptoms had started to appear, i.e. when she was 7 years old. Prior to MONA spectrum disorder a 3-methyl-crotonyl-CoA-carboxylase deficiency (3-MCC deficiency) had already been diagnosed in this patient. This is a rare autosomal recessive disorder of L-leucine metabolism^20^ causing neurological symptoms and acute metabolic decompensation triggered by common infections. None of these symptoms had ever been noted in patient 1. However based on published data showing that only less than 10% of the affected individuals ever develop minor symptoms and only less then 1% to 2% might be at risk for severe adverse outcome^21^. None of the patients with 3-MCC deficiency is reported to have a skeletal phenotype. Still, it was briefly thought to be the cause of the skeletal symptoms in Patient 1. For this reason oral L-carnitine supplementation, the recommended therapy in case of 3-MCC deficiency, was started. Dietary L-leucine restriction was not initiated as the efficacy of this approach for 3-MCC deficiency is unproven^22^.

**Figure 2:**
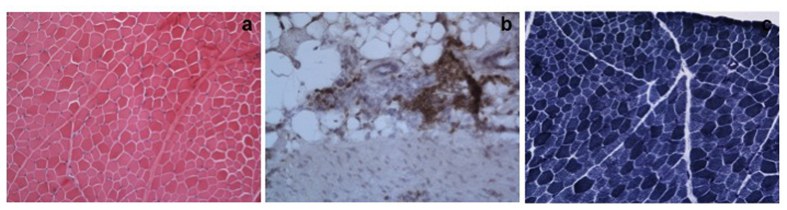
Hematoxylin and eosin stain of the muscle-tendon biopsy of patient 1 at the age of 4.5 years shows a mildly increased fiber size variability (**a**). Immuno-histochemistry using antibodies against leucocyte common antigen (LCA) revealed an accumulation of mononuclear inflammatory cells especially perivascularly at the muscle-tendon junction (**b**). NADH-TR stain indicates a regular enzyme activity, but the weakly stained type II fibers are smaller than the more intensely stained type I fibers (**c**); original magnifications x20.

As Patient 1 also showed signs of myopathy and neuropathy with reduced muscle strength and deep tendon reflexes, a muscle-tendon biopsy (musculus gastrognemicus/Achilles tendon) was performed at the age of 6 years. It demonstrated mild muscle fibre atrophy (Fig. 2a) and an accumulation of mononuclear inflammatory cells, especially around vessels within the fat connective tissue at the muscle-tendon junction (Fig. 2b). The enzyme histochemical stain for nicotinamide adenine dinucleotide-tetrazolium reductase demonstrated regular enzyme activity, but slight atrophy of type II fibres (Fig. 2c). Thus, additionally to oral L-carnitine supplementation, immunosuppressive therapy with azathioprine (2 mg/kg) was commenced. Both L-carnitine supplementation and azathioprine therapy were discontinued after the diagnosis of MONA spectrum disorder had been established when the patient was 7 years old.

Patient 2, currently 9 years old, and Patient 3, currently 7 years old, had a similar history. Both younger siblings experienced first symptoms at the age of 2 years. Clinical examination revealed decreased range of motion in the fingers, wrists, ankles and toes with contractures of the interphalangeal joints. In Patient 2 we additionally diagnosed an iris coloboma and a ventricular septal defect, which were treated conservatively. Patient 3 had a secundum atrial septal defect and a vesicoureteral reflux II°–III°, without any episodes of recurrent urinary infections. The diagnosis of MONA spectrum disorder was genetically confirmed when the patients were 7 years (Patient 2) and 5 years (Patient 3) old respectively.

All three patients received concomitant physical and occupational therapy from when first symptoms of MONA spectrum disorder appeared. Before initiation of bisphosphonate therapy all three patients needed regular analgesic therapy with non-steroidal anti-inflammatory drugs three times a day.

All three patients were found to carry the same homozygous mutation c.[1699C > T] in exon 11 of the *MMP2* gene. This mutation had not been reported previously but is very likely to be pathogenic as it leads to the introduction of a premature stop codon, p.[Arg567Ter], and early truncation of the MMP2 protein. The same mutation was then identified in heterozygosity in the healthy brother as well as in the parents.

### Bisphosphonate therapy

In Patient 1 bisphosphonate therapy was started already 3 years before the diagnosis of MONA spectrum disorder was established, i.e. when Patient 1 was 4 years old. She received intravenous pamidronate (1 mg/kg/d on two consecutive days every 3 months) for the treatment of osteolysis and osteopenia for eight consecutive years. Then, for convenience of the patient and because reported to be equally effective, the therapy was switched to intravenous zoledronate (0.05 mg/kg/d in a single dose), which in contrast to pamidronate has to be given not every 3 month but only every 6 month.

Patients 2 and 3 were started to treat with zoledronate after the diagnosis of MONA spectrum disorder had been genetically confirmed, which was at the age of 7 and 5 years respectively, i.e. 5 and 3 years after first clinical symptoms had appeared.

### Disease progression and therapeutic success

All three patients had normal developmental milestones, were of normal intelligence and frequented regular school. During follow-up no particular internal medical problems were noted. Patient 1 transiently showed mild signs of myopathy and neuropathy, which resolved spontaneously. The other siblings did not show any neurological symptoms.

**Figure 3:**
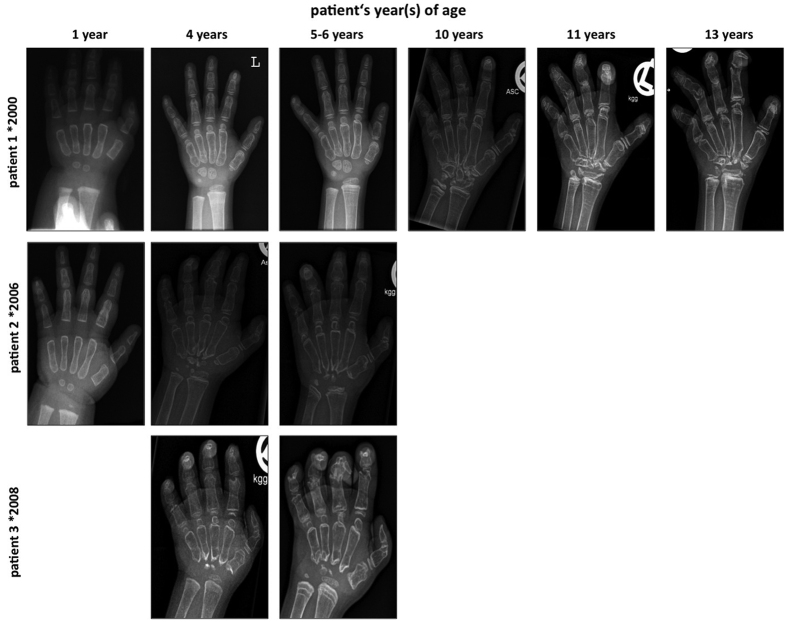
Dorsopalmar radiographs of the left hand demonstrate severe general osteopenia, progressive destruction of carpal bones and joints, multiple osteolyses, widening of phalanges and metacarpals with thinning of cortical bone and irregular epiphyses. Pronounced sklerotic metaphyseal lines most evident at the radial and ulnar bone are due to bisphosphonate therapy.

**Figure 4:**
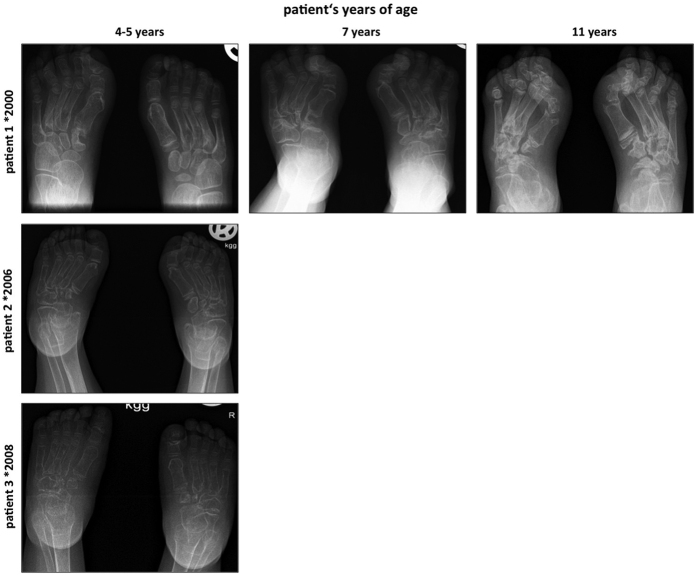
Dorsoplantar radiographs of the feet show general osteopenia, progressive joint destruction and osteolyses most pronounced at the tarsal bones. Patients 2 and 3 have more severe findings compared to patient 1.

The main sequealae for the patients were the evolving orthopaedic problems related to MONA spectrum disorder. All three patients showed short stature. In Patient 1 joint destruction progressed towards proximal to involve the wrists, elbows and knees, resulting in flexion contractures. Orthopaedic surgical treatment of a radial head dislocation was necessary. Details on this orthopaedic procedure have already been reported before in the study of AlKaissi *et al.*, were Patient 1 corresponds to Patient III^23^. Patient 2 and Patient 3 even had a more severe evolution in terms of joint destruction and flexion contractures as documented by radiographs of hands and feet, which demonstrate general osteopenia, multiple osteolysis, widening of the phalanges and irregular epiphyses (Figs 3 and 4) but no low impact fractures. Still, all three patients depended on regular analgesic therapy with non-steroidal anti-inflammatory drugs three times a day. Physical and occupational therapy were only slightly able to ameliorate skeletal pain in the patients.

**Figure 5:**
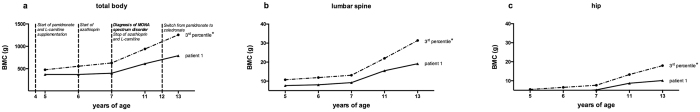
Bisphosphonate therapy increased overall bone mineral content (**a**), as well as bone mineral content in the axial (**b**) and appendicular skeleton (**c**). Bone mineral content was below the 3rd percentile at all measured time points but increase was steady and similar to age, sex and ethnicity matched healthy controls^24^ *. Data are given exemplarily for patient 1 including therapeutic interventions (**a**). Graphs demonstrate bone mineral content.

Bisphosphonate therapy significantly reduced skeletal pain in our patients. Frequency of need for analgesic therapy diminished to a single therapeutic dose 3–4 times a month. Bone densitometry demonstrated that bisphosphonate therapy led to a steady increase in bone mineral content both in the axial and in the appendicular skeleton in all three patients. Bone mineral content was below the 3^rd^ percentile at all measured times. It showed a steady increase, with a curve similar to that for age, sex- and ethnicity-matched healthy controls, although the discrepancy appeared to slightly grow with age^24^. Figure 5 exemplarily demonstrates data for Patient 1, as she has the longest observation period (8 years so far) (Fig. 5a–c). No further low-impact fractures occurred after starting bisphosphonate therapy. Patient 1 showed the best outcome. Although the nature of the disease is progressive, she is still able to walk with aids and use her hand for writing.

Patients 2 and 3 have a more severe disease course. Although they are pain-free, they are severely impaired in their daily life. This impairment primarily results from impaired movement of wrist and finger joints as well as from the fact that both siblings are wheelchair bound.

No adverse effects of bisphosphonate treatment, such as severe hypocalcaemia or nephrocalcinosis, were noted during this treatment period of ten years.

## Discussion

In this study we report successful bisphosphonate therapy in three siblings affected from MONA spectrum disorder.

MONA spectrum disorder is characterized by short stature, severe joint contractures, peripheral corneal opacities, coarse face, osteolysis of carpal and tarsal bones as well as generalized osteopenia^25^. Characteristics of patients reported in the literature are summarized in Table 1 (Table 1). Only patients with a genetically confirmed diagnosis were included in the table. The underlying cause of MONA spectrum disorder is a mutation in the gene encoding MMP2^4^,^7^.

The three sisters reported here presented with typical clinical findings and were diagnosed as having MONA spectrum disorder based on the molecular analysis revealing a novel stop mutation in the *MMP2* gene. From the genetic findings we strongly suspect that the patients’ parents are consanguineous.

Patient 1 was identified to additionally have a 3-MCC deficiency. This was already reported in the study of AlKaissi *et al.*, were Patient 1 corresponds to Patient III^23^. It is the first time that an inborn disorder of metabolism was observed in a patient with MONA spectrum disorder (Table 1). However, the association is likely to be fortuitous.

Patients 2 and 3 both show additional cardiac manifestations, i.e. a VSD and an ASD. This emphasizes a possible role of *MMP2* in the pathophysiology of cardiac pathologies suggested by experimental studies^26^,^27^,^28^, especially as cardiac manifestations have already been reported in several patients with MONA spectrum disorder^5^,^9^.

Little information on the treatment of MONA spectrum disorder is given in the literature. Controversial data exist regarding immunosuppressive treatment. Al-Mayouf *et al.* reported an inhibitory effect of methotrexate combined with D-penicillamines on progression of joint contractures^29^. Tuysuz *et al.* noted an improvement in contractures in only one patient under prednisolone therapy, but not in his similarly affected cousin^30^.

In our patients we detected significant clinical improvement under bisphosphonate therapy. Bisphosphonates decrease osteoclastic activity, thereby favouring bone formation^31^. In paediatric patients they are used to treat conditions like osteoporosis and osteogenesis imperfecta. These agents have been reported to have analgesic effects, improve range of movement, function and bone mineral content^32^. With bisphosphonate therapy we were able to reduce the need for non-steroidal analgesics from three times a day to 3–4 times a month in our patient, which dramatically improved their quality of life. Bisphosphonate therapy was not able to significantly improve range of motion. Still, one of the reported patients is able to walk with aids at the age of 14 years. This has been reported only in three other adult patients by Azzollini *et al.*^1^ and Jeong *et al.*^6^, which again underlies the broad inter- and intrafamilial variability of MONA spectrum disorder. We are also aware that our study suffers from the limitation of a retrospective design.

Bisphosphonate therapy also led to an increase in bone mineral content in the axial and the appendicular skeleton in all three patients. Bone mineral content in the patients was always below the 3^rd^ percentile, but increase rate was similar to that of age-, sex- and ethnicity-matched healthy controls (Fig. 5), although the discrepancy with these controls appeared to slightly grow with age regardless of the site of measurement. The reason is unclear. We hypothesize that it might be related to a decreasing level of physical activity of the patients as the disease progresses. It might also be related to the changing hormonal regulation during puberty, which eventually has a negative impact on bone mineral density in patients with MONA spectrum disorders. However this is highly speculative and further studies will be needed to clarify this point.

After initiation of bisphosphonate treatment no further low-impact fractures occurred in the herein reported patients. Effects were similar for both agents used (pamidronate and zoledronate). We do not attribute the more favourable clinical outcome of patient 1 to the fact that she was initially treated with pamidornate while treatment in the siblings was started with zoledronate. We hypothesize that the more benign course might be related to early initiation of bisphosphonate therapy in this patient (one year after appearance of first clinical symptoms). In contrast in Patient 2 and 3 bisphosphonate therapy was only started 5 and 3 years respectively after clinical symptoms had appeared. Furthermore Patient 1 developed first symptoms of MONA spectrum disorder at the age of 4 years whereas Patient 2 and 3 showed first symptoms already when they were 2 years old. This reflects intrafamilial variability in the disease course with siblings 2 and 3 being more severely affected. Also we do not suspect that the favourable course of Patient 1 was due to the initial combination of immunosuppressive therapy with azathioprin and bisphosphonate therapy. This hypothesis is supported by the fact that also the two other reported sisters, who haven’t had immunosuppressive treatment, clinically improved after bisphosphonate therapy was started. Furthermore MMP2 has not been reported to trigger auto-inflammatory cascades, thus patients with MONA spectrum disorder might hardly benefit from immunosuppression^33^.

Besides our study there are two more reports on the use of bisphosphonates for the treatment of MONA spectrum disorder. Phadke *et al.*^19^ administered intravenous pamidronate in a dose of 1 mg/kg/d on three consecutive days at four-monthly intervals for 3 years in two siblings affected with MONA spectrum disorder. Similarly to our study they noticed a subjective decrease in pain, tenderness and joint contractures at the wrist as well as increase of bone mineral density of the axial skeleton but no increase at the radius and ulna. Al-Mayouf *et al.*^34^ treated seven children with NAO syndrome with intravenous pamidronate. The dosis was 2 mg/kg on each of three consecutive days every three month for one year. The patients were maintained on 800 IU/day vitamin D and at least 800 mg/day of elemental calcium supplement. The authors also noticed a decrease in limb and joint pain and improvement in functional ability although not significant. However bone mineral density had increase significantly in all patients at the end of treatment^34^.

Promising results of bisphosphonate therapy have been already reported multiple times in other inherited osteolysis syndromes in children, i.e. inherited multicentric osteolysis^35^. Three siblings were treated with intravenous pamidronate at an initial dose of 0.5 mg/kg, which was increased to 1 mg/kg administered monthly for the next six months. Pain improved dramatically, no new fractures or focal osteolytic areas occurred. Similar to our patients, range of motion did not improve. Positive effects of bisphosphonates were also observed in patients with complex regional pain syndrome (CRPS), especially if they had osteopenic or osteoporotic changes in the affected limb. Amelioration of symptoms was observed both in patients who were treated orally (eg. alendronate) and intravenously (eg. clodronate or pamidronate)^36^,^37^,^38^,^39^,^40^. However the positive effects of bisphosphonates in CRPS are probably not related to their antiresorptive properties but to the fact that they might be able to modulate various inflammatory mediators that are up-regulated in CRPS-I^40^. This fact might also contribute to the positive effect of bisphosphonates observed in the treatment of MONA spectrum disorder.

## Conclusion

From the presented data we conclude that bisphosphonates are a suitable treatment option to reduce skeletal pain thereby increasing quality of life in patients with MONA spectrum disorder. Early initiation of therapy, i.e. when first symptoms of the disease appear seems to be particularly important to achieve the most favourable outcome. From our experience we suggest that bisphosphonates are a treatment option for patients suffering from a MONA spectrum disorder. Although range of motion does not improve, bisphosphonate therapy seems to be able to reduce skeletal pain in these patients.

## Methods

### Assessment of the patients

After informed consent had been obtained from the parents we assessed the patients regarding the following characteristics: consanguinity, clinical symptoms at diseases on-set, age at on-set of symptoms and age at diagnosis, cognitive development, progression of clinical symptoms related to the diagnosis, molecular investigations, associated disorders as well as therapies besides bisphosphonate therapy. Informed consent from the parents was also obtained to publish the patient’s photographs. All investigations were performed according to the relevant ethical guidelines. The experimental protocols were approved by the Department of Pediatrics of the Medical University of Innsbruck, Austria. The trial was registered in the registry and results database ClinicalTrials.gov on 1^st^ July 2016 (registration number NCT02823925).

### Bisphosphonate therapy

The reported patients received intravenous bisphosphonate therapy either with pamidronate (1 mg/kg/d on two consecutive days every 3 months) or zoledronate (a single dose of 0.05 mg/kg/day every 6 month).

### Evaluation of disease progression and therapeutic success

To assess both progression of MONA spectrum disorder and therapeutic success the patients were regularly evaluated clinically in 3 to 6 month intervals. Clinical evaluation comprised an internal, neurological and orthopaedic status as well as a general assessment of neurocognitive function. Additionally, need for oral analgesic therapy was documented. In irregular intervals, depending also on the clinical symptoms, x-rays of hands and feet were taken and a densitometry of the total body, lumbar spine and hip was performed.

## Additional Information

**How to cite this article**: Pichler, K. *et al.* Bisphosphonates in multicentric osteolysis, nodulosis and arthropathy (MONA) spectrum disorder – an alternative therapeutic approach. *Sci. Rep.* **6**, 34017; doi: 10.1038/srep34017 (2016).

## Figures and Tables

**Table 1:** Clinical presentation and *MMP2* mutations in patients with MONA spectrum disorder (formerly named nodulosis, arthropathy and osteolysis (NAO) syndrome, Winchester, Torg or Torg-Winchester syndrome) reported in the literature including our patients.

	Martignetti *et al.*^7^		Zankl *et al.*^10^	Rouzier *et al.*^8^	Zankl *et al.*^11^		Tuysuz *et al.*^30^	Gok *et al.*^5^	Jeong *et al.*^6^	Temtamy *et al.*^9^		Castberg *et al.*^2^	Ekbote *et al.*^3^	Azzollini *et al.*^1^		**Our study**
origin	3 families from Saudi Arabia, no MMP2 mutation detected in family 2		Southern Italy	Algeria	n.i.		Turkey	n.i.	Korea	Egypt		Morocco	Indian	2 Italian siblings		Turkey
consanguinity	yes		yes	yes	n.i.		yes	yes	no	yes		yes	no	yes		**probable**
**Diagnosis**	NAO		Winchester	Winchester	Torg		MONA	Torg-Winchester	Torg-Winchester	Torg-Winchester		MONA	Torg	MONA		**MONA**
**Clinical and radiological features**	family 1	family 3								family 1	family 2			patient II-1	patient II-3	
osteolysis																
carpal and tarsal	+++	+++	+++	+++	+++		++	+++	+++	+++	+++	+++	+++	+++	+++	**+++**
other bones	++	++	++	++	++		+	++	+++	+++	+++		++	++	++	**++**
wide metacarpals	yes	yes	no	no	yes		yes	yes	no	no	no	no	yes	n.i.	n.i.	
osteoporosis	+++	+++	+++	+++	+++		+++	+++	+++	+++	+++	+++	+++	+++	+++	**+++**
subcutaneous nodules	yes	yes	no	no	yes		yes	yes	no	yes	yes	yes	yes	yes	yes	**no**
facial dysmorphism	yes	yes	slightly	slightly	no		yes	no	no	yes	yes	yes	yes	slightly	slightly	**yes**
skin lesions/hirsutism	yes	yes	yes	in childhood (1/2)	yes		yes	no	yes	yes	yes	no	yes	yes	yes	**yes**
ocular defects	no	no	no	no	no		yes	chronic papilledema	no	no	no	no	no	bilateral pterygium	no	**iris coloboma**
intelectual disability	no	no	no	no	no		no	no	no	no	no	no	no	no	no	**no**
cardiac manifestations	no	no	no	no	no		BAV, TGA	ASD II	no	no	MVP	DORV, VSD, CoA	no	AV block I°	early-onset arterial hypertension	**VSD/ASD II**
other manifestations/co-morbidities	no	no	colloid nodular goitre, DM I	no	no		no	no	no	polycystic ovaries	no	no	premature thelarche	no	no	**3-MCC-deficiency, omphalocele/**
**VUR**																
age of diagnosis	n.i.	8 m/1 y	2 y	6 m/3 y	8 m		6 m/3 m	5 y/6 y	3 y	2 y/4.5 y	4 y	5 y	3 y	infancy	3 y	**4 y/7 y/5 y**
age at description	n.i.	4/20 y	21 y	24 y/35 y	9 y		6 y/4 y	13 y/11 y	31 y	17 y/16 y	10 y	8 y	n.i.	43 y	37 y	**14 y/8 y/6 y**
**Mutation**	**MMP2**	**MMP2**	**MMP2**	**MMP2**	**MMP2**	**MMP2**	**MMP2**	**MMP2**	**MMP2**	**MMP2**	**MMP2**	**MMP2**	**MMP2**	**MMP2**	MMP2	**MMP2**
	family 1	family 3			2 mutations in the same patient											
exon	2	5	8	8	2	8	11	4	8	n.i.	3	n.i.	n.i	8	8	11
nucleotide change	c.591G>A	c.1021C>A	c.1210G>A	c.1488_1490delTGG	c.302G>A	c.1357delC	c.1732delA	c.658+2T>C	c.1217G>A	c.540T>G	c.452G>A	c.301C>T	c.538G>A	c.1228G>C	c.1228G>C	**c.1699C>T**
amino-acid change	p.R101H	p.Y224X	p.E404K	p.V400del	p.R101H	p.1957delC	n.i.		p.G406D	pAsp180Glu	p.W151X	p.R101C	p.D180N	p.G410R	p.G410R	**p.R567X**
type of mutation	missense	nonsense	missense	deletion	missense	nonsense	frameshift		missense	missense	nonsense	missense	n.i.	missense	missense	**stop**
protein domain	prodomain	first fibronectin type-II domain	catalytic domain	catalytic domain	prodomain	before hemopexin domain	hemopexin domain		catalytic domain	n.i.	n.i.	prodomain	n.i	catalytic domaine	catalytic domaine	hemopexin domain

Abbreviations used in the Table: ASD = atrial septal defect, AV block I° = first-degree atrioventricular block, BAV = bicuspid aortic valve, CoA = coarctatio of the aorta, DM I = diabetes mellituy type I, DORV = double outlet right ventricle, m = month, MVP = mitral valve prolaps, n.i. = not indicated in the manuscript, TGA = transposition of the great arteries, VSD = ventricular septal defect, VUR = vesicoureteral reflux, y = year.
